# Length and GC Content Variability of Introns among Teleostean Genomes in the Light of the Metabolic Rate Hypothesis

**DOI:** 10.1371/journal.pone.0103889

**Published:** 2014-08-05

**Authors:** Ankita Chaurasia, Andrea Tarallo, Luisa Bernà, Mitsuharu Yagi, Claudio Agnisola, Giuseppe D’Onofrio

**Affiliations:** 1 Genome Evolution and Organization – Dept. Animal Physiology and Evolution, Stazione Zoologica Anton Dohrn, Villa Comunale, Napoli, Italy; 2 Campus UAB - CRAG Bellaterra - Cerdanyola del Vallès, Barcelona, Spain; 3 Molecular Biology Unit, Institut Pasteur de Montevideo, Montevideo, Uruguay; 4 Faculty of Fisheries, Nagasaki University, Bunkyo, Nagasaki, Japan; 5 Department of Biological Sciences, University of Naples Federico II, Napoli, Italy; National Center for Biotechnology Information, United States of America

## Abstract

A comparative analysis of five teleostean genomes, namely zebrafish, medaka, three-spine stickleback, fugu and pufferfish was performed with the aim to highlight the nature of the forces driving both length and base composition of introns (i.e., bpi and GCi). An inter-genome approach using orthologous intronic sequences was carried out, analyzing independently both variables in pairwise comparisons. An average length shortening of introns was observed at increasing average GCi values. The result was not affected by masking transposable and repetitive elements harbored in the intronic sequences. The routine metabolic rate (mass specific temperature-corrected using the Boltzmann's factor) was measured for each species. A significant correlation held between average differences of metabolic rate, length and GC content, while environmental temperature of fish habitat was not correlated with bpi and GCi. Analyzing the concomitant effect of both variables, i.e., bpi and GCi, at increasing genomic GC content, a decrease of bpi and an increase of GCi was observed for the significant majority of the intronic sequences (from ∼40% to ∼90%, in each pairwise comparison). The opposite event, concomitant increase of bpi and decrease of GCi, was counter selected (from <1% to ∼10%, in each pairwise comparison). The results further support the hypothesis that the metabolic rate plays a key role in shaping genome architecture and evolution of vertebrate genomes.

## Introduction

Most of the DNA stored in eukaryotic cells is non-coding. Long considered as “junk DNA” because of the unclear biological significance [1], a rising body of evidence “sound the death knell” for the concept of useless DNA [2]. The results, mainly produced in the last decade, are clearly showing that non-coding regions are involved in replication and transcription functions [3]–[7].

In spite of the increasing evidence supporting an “abundant purifying selection” for intronic regulatory sequences [8], the forces driving the evolution of the intron architecture (namely length and base composition) still remains a debated subject. The link between intron length and base composition (the molar ratio of guanine plus cytosine, i.e. GC content) was first observed by Duret and colleagues analyzing several vertebrate genomes [9]. These authors reported that not only the coding sequences, but also the corresponding intronic sequences where shorter in the GC-rich genes [9]. Interestingly, the different intron length of GC-poor and GC-rich genes was not affected by the occurrence of repetitive and/or transposable elements [9]. Further analysis carried out at the intra-genome level linked intron length to gene expression. On one side, several authors pointed out that small introns were selected for highly expressed genes, thus favoring the hypothesis based on selection for transcription efficiency and/or economy [10]–[16]; on the other, a selection for the compactness of housekeeping genes was pointed out, thus upholding the hypothesis of a genome design [17]. However, more detailed analysis revealed that: i) housekeeping genes were no more compact than the narrowly expressed genes [15], and ii) a higher occurrence of short intron sequences in GC-rich more than in GC-poor genes was highlighted by several and different statistical approaches [13], [17]–[19].

In 1995, Hughes and Hughes [20], comparing introns sizes in human versus chicken, noted that introns were shorter in birds, an observation confirmed later on by more exhaustive studies [21], [22]. The authors pointed out that evolutionary constraints linked to the metabolic cost of flight, probably would shape the intron size [20]. A hypothesis, indeed, supported by the observation that the basal mammalian metabolic rate was lower than the avian ones [23].

A comprehensive analysis on a large dataset of fish genomes showed that not only the routine metabolic rate (temperature-corrected by Boltzmann's factor) was affected by the living habitat of the species, but also the genomic GC content, both decreasing from polar to tropical environment [24]. A more detailed analysis showed that the variability of the GC content among fishes living in different habitat was not dictated by a dissimilar rate of the methylation-deamination process of the CpG doublets [25]. Between metabolic rate and GC content a significant positive correlation was found [24].

In the present paper the genomes of five fishes, namely *Danio rerio* (zebrafish), *Oryzias latipes* (medaka), *Gasterosteus aculeatus* (three-spine stickleback), *Takifugu rubripes* (fugu) and *Tetraodon nigrovirids* (pufferfish) were analyzed in the context of a link between the following variables: intron length, GC-content and metabolic rate. The results support the key role played by the metabolic rate in shaping architecture and base composition of intronic sequences.

## Materials and Methods

Coding sequences (CDS) of the genome assembly were retrieved from the ENSEMBL (http://ftp.ensembl.org) for all five fishes namely:

***D. rerio*** (Assembly: Zv7, Apr 2007, Ensembl Release: 48.7b); ***O. latipes*** (Assembly: HdrR, Oct 2005, Ensembl Release 48.1d); ***G. aculeatus*** (Assembly: BROAD S1, Feb 2006, Ensembl Release 48.1e); ***T. rubripes*** (Assembly: FUGU 4.0, Jun 2005, Ensembl Release 48.4h); ***T. nigroviridis*** (Assembly: TETRAODON 7, Apr 2003, Ensembl Release 48.1j).

Intronic sequences were retrieved from UCSC Genome browser (http://genome.ucsc.edu), for all five fishes namely: ***D. rerio*** (Assembly: Apr 2007, Zv7/danRer5); ***O. latipes*** (Assembly: Oct 2005, NIG/UT, MEDAKA 1/ oryLat2); ***G. aculeatus*** (Assembly: Feb 2006, BROAD/gas Acu1); ***T. rubripe*** *s* (Assembly: Oct 2004 (JGI 4.2/ fr2); ***T. nigroviridis*** (Assembly: Feb 2004, Genoscope 7.0/tetNig1). In each genome the number of full length genes (i.e. CDS + introns) was: *D. rerio* 17085, *O. latipes* 13247, *G. aculeatus* 16101, *T. rubripes* 19123, *T. nigroviridis* 10898. Sequences containing ambiguity in identification of certain bases were discarded. Basic sequence information were retrieved by using Infoseq, an application of EMBOSS package (EMBOSS, Release 5.0; http://emboss.sourceforge.net/). The software CodonW (1.4.4) was used to detect stop codons within the reading frame of CDSs (hence removed from the dataset before inferring orthology) and to calculate the molar ratio of guanine plus cytosine (GC).

Orthologous CDS were identified using a Perl script, which performs reciprocal Blastp [26] and selects the Best Reciprocal Hits. The e-value threshold to filter the blast results was e^−10^. Once pairs of orthologous CDS were identified between two species, the orthology was extended to the corresponding intronic sequences. More precisely, if the coding sequence *j_ith_* of species *m* (CDS*_jm_*) turned out to be the ortholog of the coding sequences *k_ith_* of species *z* (CDS*_kz_*), the intronic sequence (i.e. the sequence obtained concatenating all internal introns) of CDS*_jm_* was considered ortholog to the intronic sequence of CDS*_kz_*. Introns at 5′- and 3′-flanking regions were disregarded. The differences in GC-content (ΔGCi) and length (Δbpi) of intronic sequences were computed for each pair of orthologs. Sequences showing ΔGCi<|0.1%| and/or Δbpi<|100| were disregarded from further analysis. The histogram showing the percentage of sequences removed in each pairwise comparison, before and after removing repetitive and transposable elements, was reported as supplementary material (Figure S1). Incidentally, the amount of sequences removed was well below the threshold of 10%, unless in the comparison *T. rubripes vs. T. nigroviridis* (∼20%), essentially due to the very short phylogenetic distance between the two species [27].

The number of orthologous intronic sequences in each of the ten possible pairwise combinations among the five fishes were the following: *D. rerio - O. latipes (*2874*); D. rerio - G. aculeatus (*5703*); D. rerio - T. rubripes (*5351*); D. rerio - T. nigroviridis (*4473*); O. latipes - G. aculeatus (*3206*); O. latipes - T. rubripes (*2822*); O. latipes - T. nigroviridis (*2583*); G. aculeatus - T. rubripes (*5966*) G. aculeatus - T. nigroviridis (*5077*); T. rubripes - T. nigroviridis (*4401*).* The percent of positive ΔGCi was calculated as follows:
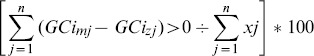
where: *n* is number of orthologous genes between two species *m* and *z*. The percent of positive Δbpi between species m and z was calculated following the same rules. Needless to say, the percent of negative events was the complement to hundred.

RepeatMasker (Version 3.1.9, http://repeatmasker.org) was used to mask the interspersed repeats and low complexity DNA sequences.

Statistic was performed using the software StatView 5.0 and the VassarStats website (http://www.vassarstats.net/index.html). Data regarding physiological and environmental parameters of the five teleostean fishes were retrieved from www.fishbase.org/.

### Specimens

Zebrafish and pufferfish were obtained from a local store (CARMAR, Italy), whereas three-spine stickleback (from now on shortly termed as stickleback) were collected in the Nature Reserve of Posta Fibreno (FR, Italy). Medaka specimens were kindly provided by Dr. Conte (IGB, Naples – Italy).

Animals were maintained in the facilities of the Dept. of Biology of the University of Naples Federico II, and were acclimated for a minimum of 14 days prior to experiments in glass tanks with dechlorinated, continuously filtered and aerated water, with 10 h:14 h L:D photoperiod. Distinct environmental parameters were set for each species, according to their habitat conditions, respectively: Zebrafish: 27°C, freshwater, pH 7.0; Medaka: 26°C, freshwater, pH 6.5 (controlled via a CO_2_ controller); Stickleback: 20°C, freshwater, pH 7.0; Pufferfish: 26°C, 10‰ salinity, pH 8.4. Zebrafish and medaka were fed daily with commercial pellet (Tetramin, Tetra, Germany). Stickleback and pufferfish were fed daily with Chironomus' larvae (Eschematteo s.r.l., Italy). All the species displayed a normal behaviour in the maintenance tanks. Before measuring oxygen consumption specimens were fasted for 48 h. The procedures described above were approved by the Animal Care Review Board of the University Federico II of Naples. Regarding fugu, data are available in [28].

### Respirometry

Oxygen consumption of individual specimen was performed in a closed system, using a respirometer whose volume was different according to the species used (ranging from 50 to 200 ml). Water conditions in the respirometer were identical to those of maintenance tanks for each species. An oxygen microelectrode (YSI 5357 Micro Probe, USA) was set through the respirometer cover to continuously record the water oxygen content. The microelectrode was connected to an Oxygen Monitor System (YSI 5300 A), whose output signal was acquired via an analogical-digital interface (Pico Technology Ltd, UK) connected to a PC for automated data acquisition using a specific software (Picolog Pico Technology Ltd., UK). Water in the respirometer was fully aerated and continuously stirred to maintain uniform the oxygen concentration. Before introducing the fish into the respirometric chamber, the oxygen sensor was calibrated at 100% air saturated water. Animals were weighed, transferred into the respirometric chamber and left undisturbed for 10–30 min to adapt to the new ambient. After adaptation, aeration was set off, the chamber was closed, and the fall in oxygen content was recorded. No more than 15–20% of oxygen content fall was allowed. Atmospheric pressure during determination was measured and used to calculate pO_2_ according to the equation:
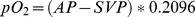
where: *AP* is the atmospheric pressure (kPa), *SVP* is the saturated vapor pressure of water at the temperature of measurement, and 0.2096 the O_2_ fraction in the air. From the pO_2_ value, the oxygen concentration, in mg l^−1^, was calculated as: [O_2_]  =  pO_2_ x α, where α (in mg-O_2_ l^−1^ kPa^−1^) is the oxygen solubility in water at the temperature and salinity of measurement. Knowing the chamber volume, the total amount of oxygen (in O_2_µg) in the chamber as a function of time during the oxygen consumption measurement is determined. The linear regression of the total oxygen *vs.* time relationship gives the amount of oxygen consumed by the animal per unit time. Dividing this value by the animal weight gives the specific oxygen consumption. Regarding fugu, Yagi and colleagues [28] followed a similar methodology, and published results were supplemented with additional data.

Data regarding oxygen consumption were obtained in resting or routine conditions, avoiding any possible source of stress. Fish mass specific metabolic rate values, expressed as mgO_2_ x kg^−1^ x h^−1^, were temperature-corrected using the Boltzmann's factor (MR = MR_0_e^E/kT,^ where MR is the temperature- corrected mass specific metabolism, MR_0_ is the metabolic rate at the temperature T expressed in K; E (energy activation of metabolic processes)  = <0.65 eV; k (the Boltzmann's constant)  = 8.62×10^−5^ eV K^−1^ [29].

## Results

**Figure 1:**
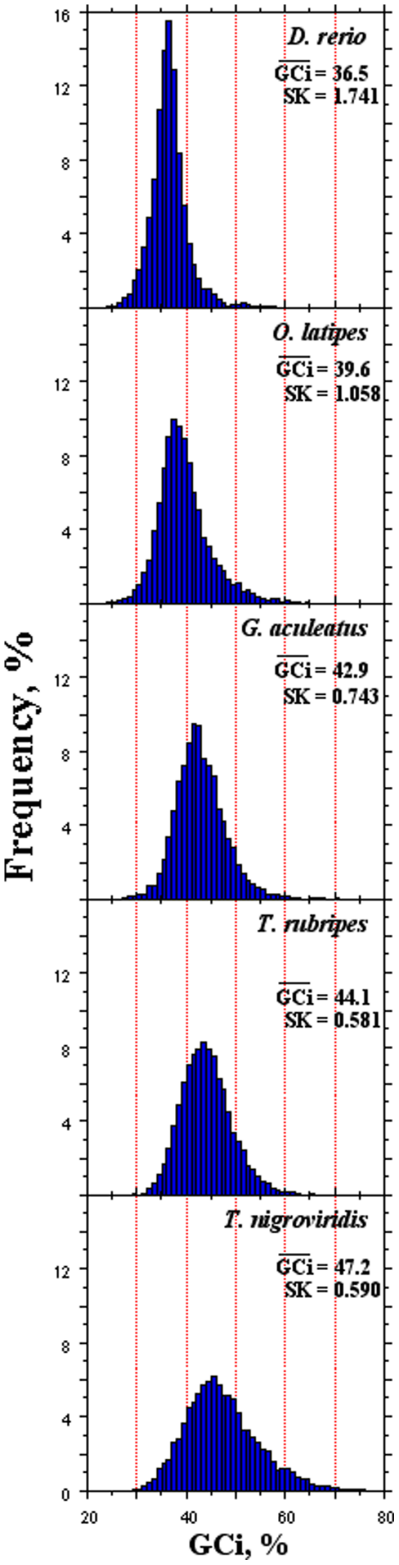
The histograms show the base composition distribution of the intronic sequences (GCi) in the five teleostean genomes. In each panel, the average GCi and the skewness (SK) of the distribution are reported.

The five species analyzed in the present paper, ordered according to the phylogenetic tree reported in [27], showed an increasing GC-content (Table 1). The genomic and the intronic base composition (GCg and GCi, respectively) showed the same ranking order, i.e. *D. rerio* (zebrafish) ***<*** *O. latipes* (medaka) ***<*** *G. aculeatus* (stickleback) **<** *T. rubripes* (fugu) ***<*** *T. nigroviridis* (pufferfish). In each species, GCi was lower than the corresponding GCg, with the exception of *T. nigroviridis*. As expected, the two variables were significantly correlated (*p*-value<6.7×10^−3^). On the contrary, bpi showed no correlation with GCg, GCi (Table 1). In Fig. 1 (panels A-E), the histograms of the GCi distribution in each genome were reported. Species were ordered according to the increasing phylogenetic distance [27]. Interestingly: i) the GCi% was higher in stickleback than zebrafish; and ii) the values of the skewness (SK) were negatively correlated with the corresponding GCi%. These results were in contradiction with the thermostability hypothesis, since GC and genome heterogeneity (due to the formation of GC-rich isochores) are expected to increase at increasing environmental temperature ([42], for a review). The complete statistical analysis of GCi distribution in each genome was reported as supplementary material (Table S1).

**Table 1:** Average values of genome (GCg) and intron (GCi) base composition, intron lenght (bpi) and metabolic rate temperature-corrected by Boltzmann's factor (MR) in fish genomes.

	*D. rerio*	*O. latipes*	*G. aculeatus*	*T. rubripes*	*T. nigroviridis*
**GCg (%)**	37.36	40.10	44.12	45.50	45.90
**GCi (%)**	36.50	39.60	42.90	44.10	47.20
**bpi**	17992.57	3109.9	5056.68	5366.9	3011.24

The lack of correlation between bpi and both GCg and GCi (Table 1) deserved further consideration. Indeed, the number of available full gene sequences (i.e. CDS+introns) was very different for each species (see Materials and Methods). In order to avoid any bias due to the size of the datasets, the comparative genome analysis was restricted to sets of orthologous intronic sequences (see Materials and Methods). Moreover, to highlight the possible effect of transposable and/or repetitive elements, the software RepeatMasker was used to clean up all the intronic sequences. The average length (bp%) of the intronic sequence masked by RepeatMasker in each species, as well as the corresponding GC%, were reported in Table 2. Regarding length, the introns of zebrafish and stickleback showed the highest and the lowest effect of the RepeatMasker step. On the average intronic sequences were shortened by a ∼6% and ∼2%, respectively (Table 2). Regarding base composition, values were increasing from zebrafish (∼14%) to pufferfish (∼42%). In spite of such a great variability, the average GCi% values before and after RepeatMasker changed slightly from set to set of orthologous introns (Table 3), and were barely different from those of the whole set of intronic sequences (Table 1). The SK values of each GCi distribution of orthologous intronic sequences, before RepeatMasker, were reported in Table S2. For each species the average SK value was: 0.45 (zebrafish), 1.087 (medaka), 0.67 (stickleback), 0.50 (fugu) and 0.69 (pufferfish).

**Table 2:** Average bp% and GC% of repetitive elements removed by Repeat Masker.

	bp%	S.E.	GC%	S.E.
***D. rerio***	5.710	0.086	14.200	0.023
***O. latipes***	2.224	0.133	23.459	0.005
***G. aculeatus***	2.040	0.032	35.517	0.003
***T. rubripes***	3.576	0.070	39.807	0.004
***T. nigroviridis***	3.059	0.058	42.685	0.004

S.E.  =  standard error.

**Table 3:** Average GCi% in each set of orthologous introns before (bRM) and after (aRM) Repeat Masker.

	*D. rerio*		*O. latipes*		*G. aculeatus*		*T. rubripes*		*T. nigroviridis*	
	bRM	aRM	bRM	aRM	bRM	aRM	bRM	aRM	bRM	aRM
***D. rerio***	-		35.12	36.55	35.38	36.43	35.38	36.5	35.41	36.53
***O. latipes***	39.52	39.67	-		39.37	39.54	39.51	39.67	39.42	39.58
***G. aculeatus***	43.32	43.40	43.01	43.09	-		43.38	43.46	43.36	43.43
***T. rubripes***	43.98	43.96	43.62	43.61	43.91	43.87	-		44.13	44.11
***T. nigroviridis***	47.06	47.14	46.42	46.49	46.88	46.96	47.09	47.16	-	

**Figure 2:**
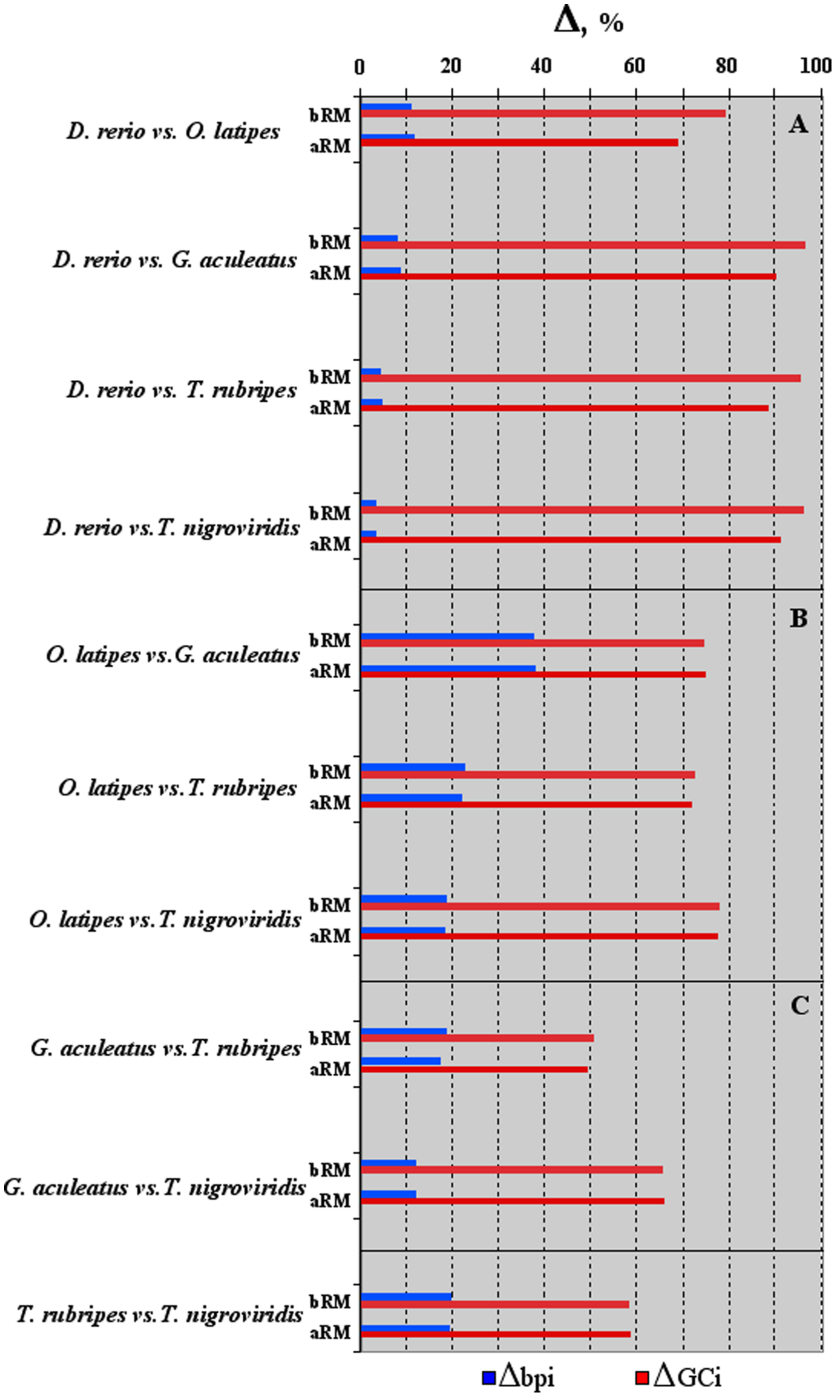
The histogram shows the percents of orthologous intronic sequences increasing in length (Δbpi, blue bars) and GC content (ΔGCi, red bars) in each pairwise comparison. Data before (bRM) and after (aRM) RepeatMasker are reported. In cluster A: comparison of medaka, stickleback, fugu and pufferfish against zebrafish. In cluster B: comparison of stickleback, fugu and pufferfish against medaka. In cluster C: comparison of fugu and pufferfish against stickleback. Within each cluster pairwise comparisons were ordered according to the increasing phylogenetic distance.

The differences in length (**Δ**bpi) and base composition (**Δ**GCi) of the intronic sequences, before and after RepeatMasker, were computed independently for each variable in each pairwise comparison of orthologous intronic sequences. The pairwise comparisons were grouped in three clusters. The first (**A**) grouping **Δ**s of medaka, stickleback, fugu and pufferfish *vs* zebrafish (i.e. **Δ**_medaka-zebrafish_; **Δ**_stickleback-_ _zebrafish_; **Δ**_fugu-zebrafish_ and **Δ**_pufferfish-zebrafish_); the second (**B**) grouping those of stickleback, fugu and pufferfish *vs.* medaka; and the third (**C**) comprising those of fugu and pufferfish *vs.* stickleback (Fig. 2). Comparisons within each cluster were ordered according to the increasing phylogenetic distance [27]. In Fig. 2, the histogram bars referred to the percentage of sequences longer (**Δ**bpi%, blue bars) and GC-richer (**Δ**GCi%, red bars) in the first of the two species (for example medaka in the **Δ**_ medaka-zebrafish_). The percents of intronic sequences longer and GC-richer in the second species (i.e. zebrafish in the **Δ**_medaka_ - _zebrafish_) accounted for the complement to hundred (not shown).

No significant differences were observed before and after RepeatMasker (Fig. 2), with the exception of data regarding cluster A, where **Δ**GCi, after removing transposable and repetitive elements, was reduced in each pairwise comparison of a ∼10%. In Fig. 2, **Δ**bpi% and **Δ**GCi% displayed an opposite behavior within each pairwise comparison, indicating that the majority of the intronic sequences were shorter and/or GCi-richer in the first of the two species (for example medaka in the **Δ**_ medaka-zebrafish_). For example, in the cluster **A**, the **Δ**bpi values, even after RepeatMasker, were very low ∼11%, ∼9%, ∼5% and ∼3%, whereas those of the corresponding **Δ**GCi were very high ∼70%, 90%, ∼88% and ∼92%. The above trend was observed also in clusters **B** and **C**, as well as in the pairwise comparison fugu *vs.* pufferfish (Fig. 2).

**Figure 3:**
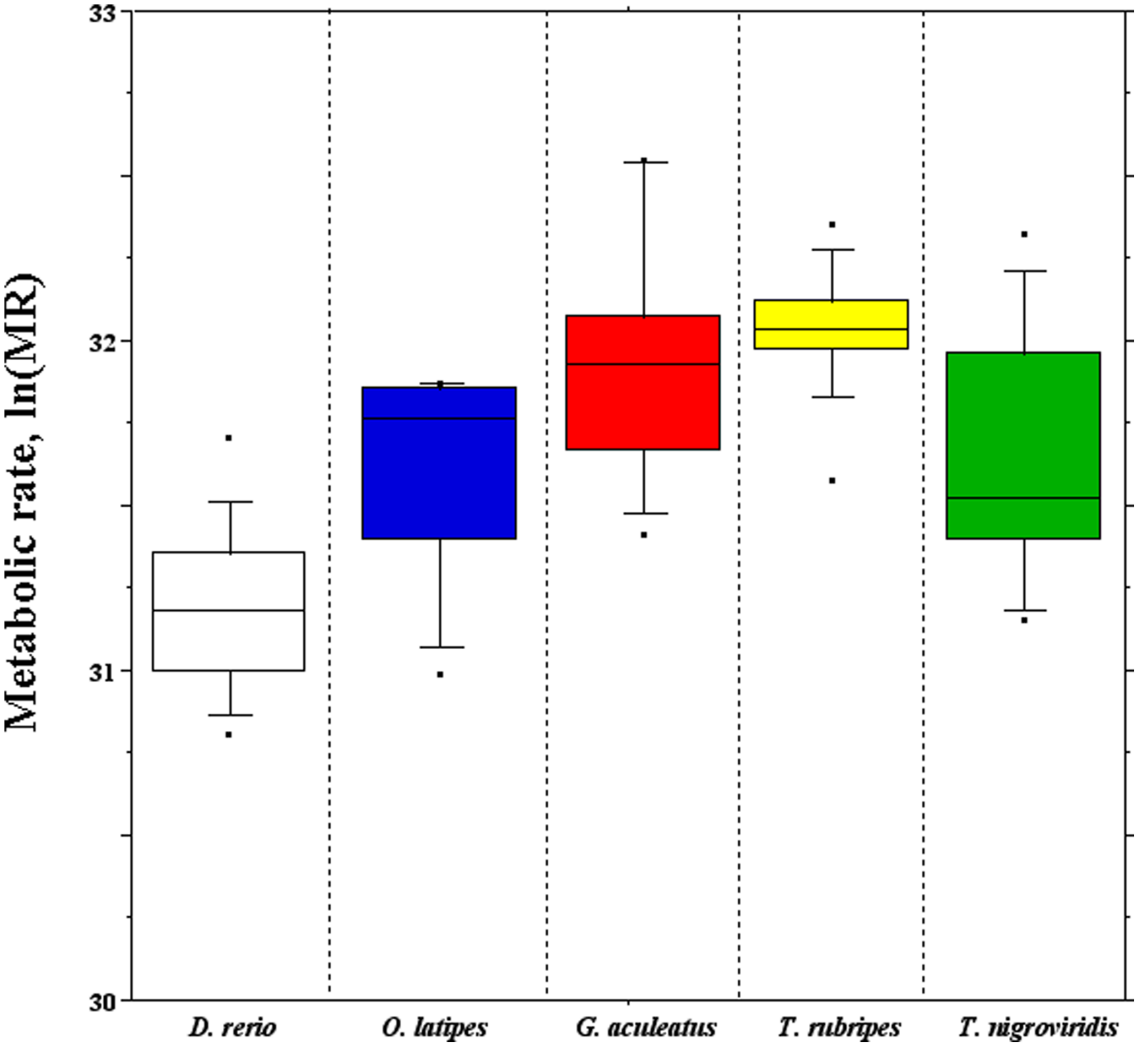
Box plots of the routine metabolic rate temperature-corrected using the Boltzmann's factor (MR) measured in each teleostean fish.

The routine metabolic rate was measured for each species. The values were temperature-corrected using the Boltzmann's factor, and shortly denoted as metabolic rate (MR). For each species, the distribution of log-normalized MR values was reported as box plots (Fig. 3), while the average values were reported in Table S3, also reporting the physiological parameters, i.e. the environmental ranges of temperature (°C) and salinity (S‰) of the five fishes. The Student-Newman-Keuls post hoc test for multiple comparisons was performed to assess the significance (threshold *p*<0.5×10^−2^) of the MR differences observed among species (Table 4). In short: i) the MR of zebrafish was significantly the lowest; ii) that of medaka was significantly lower than those of stickleback and fugu, but not significantly different from that of pufferfish; iii) the MR of stickleback and fugu were not significantly different; iv) that of pufferfish was significantly different from those of stickleback and fugu.

**Table 4:** Student-Newman-Keuls post hoc test.

	*D. rerio*	*O. latipes*	*G. aculeatus*	*T. rubripes*
***D. rerio***	-			
***O. latipes***	S	-		
***G. aculeatus***	S	S	-	
***T. rubripes***	S	S	NS	-
***T. nigroviridis***	S	NS	S	S

S  =  significant (threshold level *p*<5.0×10^−2^).

NS  =  not significant.

The MR average values showed a correlation with GCg (*p*-value<8.5×10^−2^), and no correlation with GCi. It is worth to bring to mind that in a larger dataset of 34 teleostean species the correlation between MR and GCg was highly significant, *p*-value<2.5×10^−3^ [24]. For each pair of species, the **Δ**MR values were computed and correlated with the corresponding **Δ**GCi and **Δ**bpi average values obtained before running RepeatMasker. The Spearman rank correlation test was performed to assess the statistical significance (Table 5). **Δ**GCi and **Δ**bpi were significantly correlated (Rho -0.709, *p*-value<3.3×10^−2^), as well as **Δ**GCi and **Δ**MR (Rho 0.770, *p*-value<2.1×10^−2^), while the correlation between **Δ**bpi and **Δ**MR was at the limit of the statistical significance (Rho -0.648, *p*-value<5.1×10^−2^). Replacing **Δ**MR with **Δ**T°, i.e. the increments of the average, or the maximum, environmental temperature experienced by each species, no significant correlation was observed with both **Δ**GCi (Rho -0.287, *p*-value<42.1×10^−2^ and Rho -0.126, *p*-value<72.8.1×10^−2^, respectively) **Δ**bpi (Rho -0.037, *p*-value<92.1×10^−2^ and Rho -0.101, *p*-value<78.1×10^−2^, respectively).

**Table 5:** Correlation coefficients Rho (in italic) and *p*-values (in bold) of Spearman correlation test.

	Δ bpi	Δ GCi	Δ MR
Δ bpi	**-**	**<3.3×10^−2^**	**<5.1×10^−2^**
Δ GCi	*−0.709*	**-**	**<2.1×10^−2^**
Δ MR	*−0.648*	*0.770*	**-**

Intron length (**Δ**bpi) and GC content (**Δ**GCi) were further analyzed, testing the concomitant effect of both variables on the intronic sequences. Orthologous sequences of each pairwise genome comparison were grouped into four classes, according to the following criteria:

- negative **Δ**bpi and positive **Δ**GCi values, named as N/P;
- both negative **Δ**bpi and **Δ**GCi values, named as N/N;
- positive **Δ**bpi and negative **Δ**GCi values, named as P/N;
- both positive **Δ**bpi and **Δ**GCi values, named as P/P.

**Figure 4:**
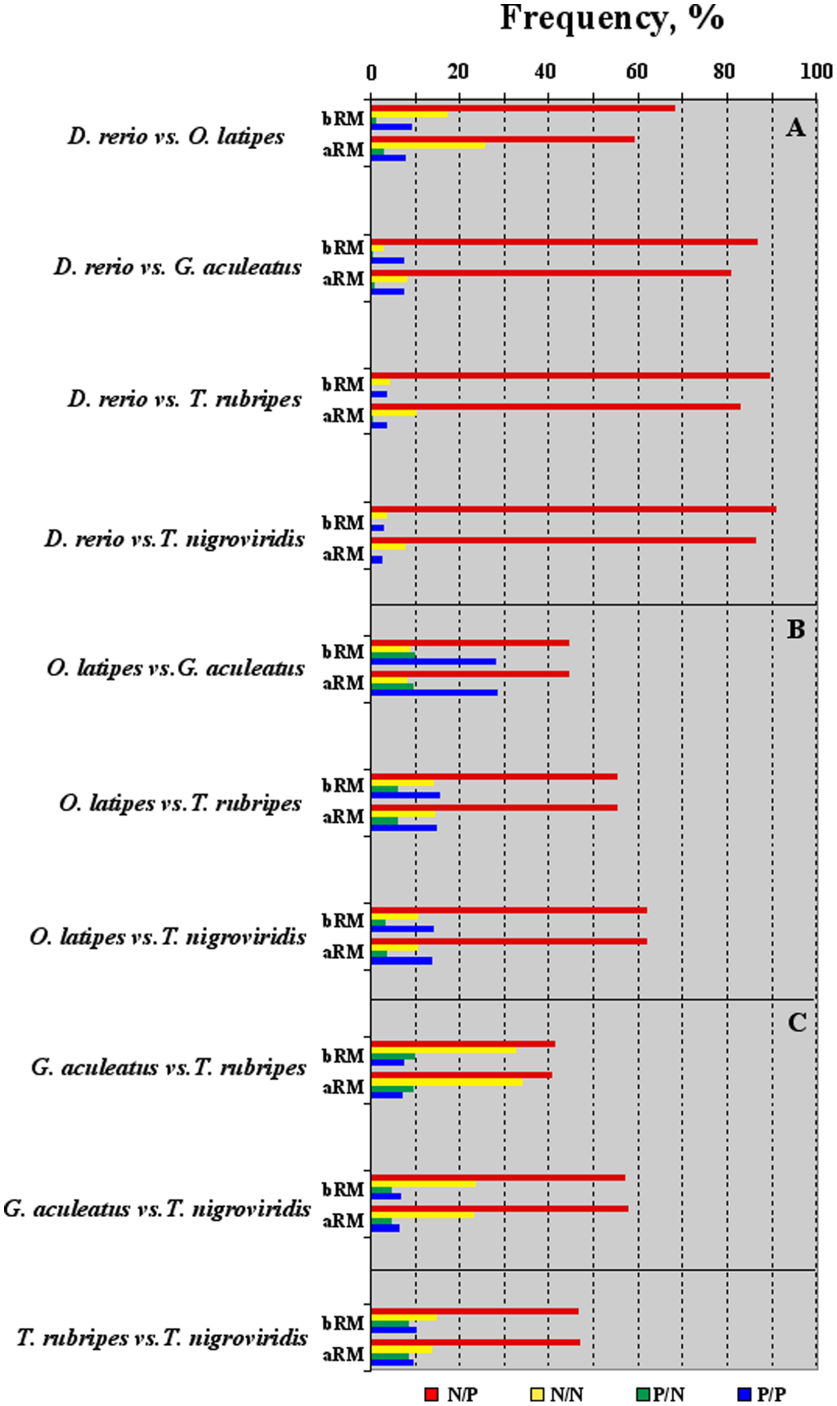
The histogram shows the percent of the four classes N/P (negative Δbpi and positive ΔGCi values), N/N (negative Δbpi and negative ΔGCi values), P/N (positive Δbpi and negative ΔGCi values) and P/P (negative Δbpi and negative ΔGCi values) in each pairwise genome comparisons. Data before (bRM) and after (aRM) RepeatMasker are reported. Clusters A, B and C as in the legend of Fig. 2.

The frequencies of each class in each pairwise comparison, before and after RepeatMasker, were reported in Fig. 4, clustered and ordered as in Fig. 2. Also in this analysis, substantial differences before and after RepeatMasker were only observed in cluster **A**, mainly affecting the N/P class (Fig. 4). Nevertheless, in all pairwise genome comparison, the N/P class showed the highest frequency. The significance of the different frequencies observed among the four classes was tested by the one-side binomial statistical test [30] (Table S4, for details). The N/P class was significantly the highest in all pairwise comparisons, *p*-value<3×10^−5^. Even after RepeatMasker, the N/P values in the cluster **A** ranged from ∼59% of **Δ**_medaka-zebrafish_ to ∼86% of **Δ**_pufferfish -_ _zebrafish_; in **B** from ∼44% of **Δ**_sticleback-medaka_ to ∼62% of **Δ**_pufferfish-medaka_; in **C** from ∼40% of **Δ**_fugu-sticleback_ and ∼58%. of **Δ**_pufferfish-sticleback_ (Fig. 4). In the comparison **Δ**_pufferfish-sticleback_ the N/P class was close to 50%.

Within each cluster, no specific trend was observed for the N/N, P/N and P/P. The N/N class was at the second rank position in six over ten pairwise comparisons, ranging from ∼3% (in zebrafish *vs.* stickleback) to >30% (in stickleback *vs.* fugu). The P/N class (ranging from <1% to ∼10%) was the less represented, particularly in cluster **A**; while the P/P class, ranging from ∼3% to ∼28%, was mainly represented in the cluster **B** (Fig. 4).

## Discussion

A general agreement on the hypothesis that selection mainly shapes the intron length through the expression level can be found in the current literature [10], [12], [13], [15], [33], [34]. On the contrary, the link between the forces shaping both the regional GC content and the intron length remains a debated issue since evidence have been produced both in favor or against [9], [13], [17], [18], [31], [33].

Within the frame of the metabolic rate hypothesis [35], Vinogradov pointed out that increments of the GC content, on one side, increase the DNA bendability [35] and, on the other, reduce the nucleosome formation potential [32]. Recently, the former point was further confirmed [36]. *In situ* hybridization of probes with different base composition showed that GC-rich chromosomal regions were, indeed, characterized by an open chromatin structure, while GC-poor ones characterized by a close chromatin structure [37]. Hence, an increment of the GC should increase: i) the probability that a GC-rich CDS, mainly bearing short non-coding sequences, could be harbored in a GC-rich and actively transcribed genome region [13], [18], [31], [38]; and ii) the DNA bendability, thus reducing the probability to have DNA breakages during the transcription process [35].

In the present study a linear correlation between intron length (bpi) and the corresponding GC content (GCi) was not found. Neither analyzing the whole data set of intronic sequences available for each genome (Table 1), nor each subset of othologous intronic sequences.

However, starting from orthologous intron sets and computing independently **Δ**bpi and **Δ**GCi in each pairwise genome comparison, a different picture came out. For example, in the pairwise comparison **Δ**_medaka-zebrafish_ the largest part of the intronic sequences of medaka showed a lower length and a higher GCi content (Fig. 2). The same applied in all pairwise comparisons before and after cleaning sequences by RepeatMasker). Differences between before and after RepeatMasker were observed only in the pairwise comparisons of the cluster **A** (Fig. 2). The effect should be ascribed to the high occurrence of type II transposable elements covering ∼39% of zebrafish genome, against a ∼10% observed in medaka, stickleback fugu and pufferfish [39].

For each species, the routine metabolic rate was measured and temperature-corrected using the Boltzmann's factor, according to [29]. Differences of the average metabolic rate (**Δ**MR) were calculated in each pairwise comparison of the teleostean species. Interestingly, **Δ**MR was correlated negatively with the average **Δ**bpi and positively with the average **Δ**GCi (Table 5) computed before RepeatMasker. Both correlations were statistically significant (Table 5). In turn, **Δ**bpi and **Δ**GCi were negatively and significantly correlated (Table 5). The correlation of **Δ**MR *vs.* **Δ**bpi was of particular interest because opened to the hypothesis that the occurrence of transposable and repetitive elements would be under the ultimate control of the metabolic rate of the organisms. A random insertion of transposable elements or a random increment of the repetitive elements in the intronic regions, indeed, should alter the opposite trend between **Δ**bpi and **Δ**GCi. However, the negative trend between the two variables was found to hold also after cleaning up intronic sequences by RepeatMasker (Fig. 2).

The analyses of the four possible combinations of the differences in intron length and GC content (the four classes in Fig. 4), further supported the inverse relationship between the two variables. Indeed, the N/P class (grouping intronic sequences showing concomitantly negative **Δ**bpi and positive **Δ**GCi values) was significantly the highest in all pairwise comparisons, *p-*value<3×10^−5^, also after RepeatMasker (Fig. 4). Conversely, the P/N class (grouping intronic sequences showing concomitantly positive values for **Δ**bpi and negative ones for **Δ**GCi) was counter selected, accounting on the average for ∼5% the orthologous set of genes.

In short, in each pairwise comparison the largest majority of intronic sequences (N/P class) were under a converging constraint for a reduction of the length and an increment of the GC content. For the other sequences grouped in the P/P, P/N and N/N classes such a converging constraint was most probably not of use, likely because of different or no constraints. Regarding the P/P and the P/N, a possible explanation could be that those classes are most probably harboring: i) genes on which the co-transcriptional splicing is taking place, a process mainly affecting genes carrying long and GC-rich introns [40]; or ii) genes showing alternative splicing, a process that was reported to be favored in genes harboring long introns [41].

A possible explanation for the discrepancy between the intra- and the inter-genomes analysis most probably could be ascribed to the fact that the former was a picture of a *status quo,* i.e. a snapshot of a genome, whereas the latter was an analysis of an *in fieri* process, i.e. a work in progress. Indeed, it is worth to recall that all pairwise comparisons between fishes were performed according to the phylogenetic relationship of the five species [27].

Recent analysis on a large dataset of fishes, ∼150 teleostean species, showed that MR and GCg were both decreasing from polar to tropical habitat and that the positive correlation between the two variables was statistically significant [24], [25].

Actually, the metabolic rate hypothesis is not the only one proposed to explain the GC content variability among and within genomes. Two alternative hypotheses have been proposed.

The first one, known as the thermodynamic hypothesis, was based on the observation that an increment of the GC content stabilizes at once DNA, RNA and protein structures against increments of temperature [42]. According to this hypothesis, increments of environmental or body temperature (for poikilotherms and homeotherms, respectively), should affect the genomic GC content, and in particular the genome base composition heterogeneity, due to the formation of GC-rich isochores [42].

Present data were not supporting the hypothesis. In fact, Jabbari and colleagues, comparing orthologous coding sequences between fugu and pufferfish, showed that both GC content and compositional heterogeneity were higher in the latter, ascribing the results to the higher environmental temperature of pufferfish [43]. However, although if our data regarding SK and GCi of these two species were in agreements with the above report (Fig. 1, Table S2 and Table 3), extending the pairwise comparisons to the other species (Table S2), discrepancies between SK, GCi and living temperature were observed. Indeed, stickleback, living in an environmental temperature range of 4–20°C (Table S3), showed both higher SK and GCi (Table S2 and Table 3) and incidentally also higher GCg (Table 1) values than those of zebrafish, living in the range of 18–24°C (Table S3). The above results, on one side, were in agreement with the structure of the isochores found in stickleback and zebrafish genomes [44], but, on the other, were in contradiction with the thermodynamic hypothesis [42]. The observation that polar teleosts were characterized by a GCg higher than those of tropical ones, not ascribed to an increased deamination process [24], [25], further confuted the thermodynamic hypothesis.

The second one, essentially based on the biased gene conversion (BGC), linked the high GC content to the high recombination rate [45]–[47]. However, an analysis of vertebrate genomes showed that no correlation was observed between GC content and recombination rate among vertebrate genomes [48]. Thus the BGC hypothesis seems not apt to explain the GC content variability among organisms. Indeed, also in bacteria the BGC hypothesis was rejected [49].

In conclusion, the metabolic rate seems to be the main selective factor driving the evolution of the genome architecture, in particular regarding length and base composition of intronic sequences. The present results not only further support previous observations about genome evolution of vertebrates [24], [25], [50], but also open a challenge for a comparative study of the gene expression level among teleosts.

## Supporting Information

Figure S1 **Removed sequences for each pairwise comparison.**
(PDF)

Table S1 **Descriptive Statistics of GCi distribution.**
(PDF)

Table S2 **Skewness of GCi% in each set of orthologous introns before RepeatMasker.**
(PDF)

Table S3 **Physiological parameters for the five analyzed fish.**
(PDF)

Table S4 **Binomial test, before and after RepeatMasker, for all the pairwise comparisons.**
(PDF)
