# Supplementary figures and images for: Length and GC Content Variability of Introns among Teleostean Genomes in the Light of the Metabolic Rate Hypothesis
Source: PLoS One. 2014 Aug 5;9(8):e103889. doi: 10.1371/journal.pone.0103889 (PMC4122358; doi:10.1371/journal.pone.0103889)

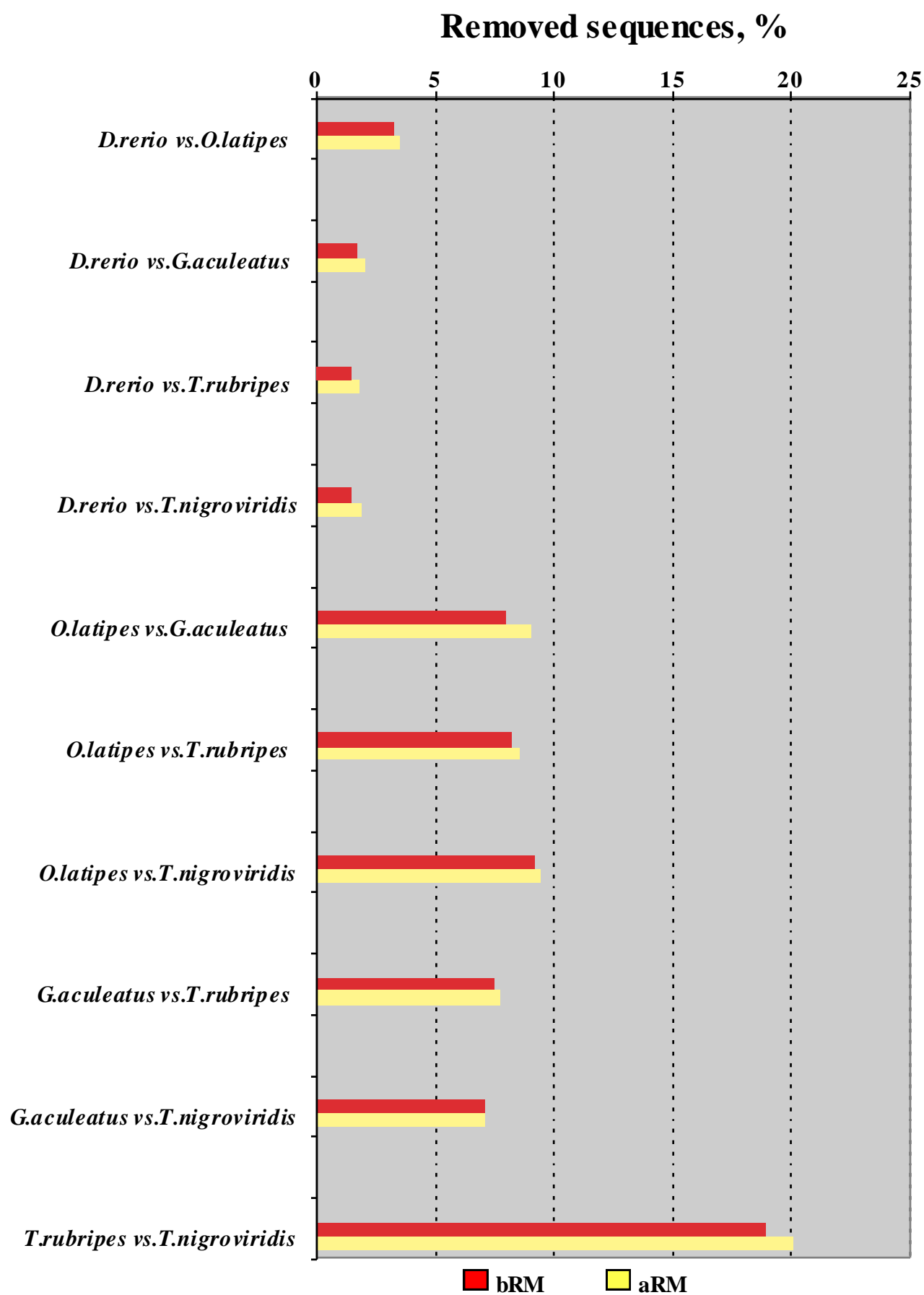

**Fig. S1**

Supplement: Figure S1 — Removed sequences for each pairwise comparison. (PDF) [file pone.0103889.s001.pdf]
