# Supplementary material for: Length and GC Content Variability of Introns among Teleostean Genomes in the Light of the Metabolic Rate Hypothesis
Source: PLoS One. 2014 Aug 5;9(8):e103889. doi: 10.1371/journal.pone.0103889 (PMC4122358; doi:10.1371/journal.pone.0103889)
Supplement: Table S1 — Descriptive Statistics of GCi distribution. (PDF) [file pone.0103889.s002.pdf]

Table S1. Descriptive Statistics of GCi distribution.

	Mean	Std. Dev.	Count	Range	Median	Skewness
D. rerio	0.365	0.041	13521	0.557	0.362	1.741
O. latipes	0.396	0.054	5779	0.455	0.388	1.058
G. aculeatus	0.429	0.052	5862	0.572	0.424	0.743
T. rubripes	0.441	0.052	13870	0.489	0.437	0.581
T. nigroviridis	0.472	0.074	8905	0.542	0.463	0.590
