# Supplementary material for: Length and GC Content Variability of Introns among Teleostean Genomes in the Light of the Metabolic Rate Hypothesis
Source: PLoS One. 2014 Aug 5;9(8):e103889. doi: 10.1371/journal.pone.0103889 (PMC4122358; doi:10.1371/journal.pone.0103889)
Supplement: Table S2 — Skewness of GCi% in each set of orthologous introns before RepeatMasker. (PDF) [file pone.0103889.s003.pdf]

Table S2. Skewness of GCi % in each set of orthologous introns before RepeatMasker

	D. rerio	***O. latipes***	***G. aculeatus***	***T. rubripes***	***T. nigroviridis***
D. rerio	-	0.473	0.375	0.457	0.499
O. latipes	1.143	-	1.114	1.094	1.054
G. aculeatus	0.602	0.656	-	0.569	0.789
T. rubripes	0.493	0.556	0.524	-	0.430
T. nigroviridis	0.579	0.753	0.674	0.648	-
