# Supplementary material for: Length and GC Content Variability of Introns among Teleostean Genomes in the Light of the Metabolic Rate Hypothesis
Source: PLoS One. 2014 Aug 5;9(8):e103889. doi: 10.1371/journal.pone.0103889 (PMC4122358; doi:10.1371/journal.pone.0103889)
Supplement: Table S3 — Physiological parameters for the five analyzed fish. (PDF) [file pone.0103889.s004.pdf]

Table S3. Physiological parameters

	lnMR	°C	S (‰)
D. rerio	31.178	18-24	0-25
O. latipes	31.587	18-24	0-35
G. aculeatus	31.937	4-20	0-35
T. rubripes	32.033	15-20	14-35
T. nigroviridis	31.637	24-28	0-35

MR = metabolic rate temperature-corrected by the Boltzmann's factor

°C and S = environmental ranges of temperature and salinity
