# Supplementary material for: Length and GC Content Variability of Introns among Teleostean Genomes in the Light of the Metabolic Rate Hypothesis
Source: PLoS One. 2014 Aug 5;9(8):e103889. doi: 10.1371/journal.pone.0103889 (PMC4122358; doi:10.1371/journal.pone.0103889)
Supplement: Table S4 — Binomial test, before and after RepeatMasker, for all the pairwise comparisons. (PDF) [file pone.0103889.s005.pdf]

TableS4. Binomial test

http://www.vassarstats.net/

Before RepeatMasker

Pairwise species	N/P	N/N	P/N	P/P
D.rerio/O.latipes	2036	519	43	276
D.rerio/G.aculeatus	5043	188	34	438
D.rerio/T.rubripes	4871	241	26	213
D.rerio/T.nigroviridis	4135	183	14	141
O.latipes/G.aculeatus	1561	312	349	984
O.latipes/T.rubripes	1702	441	197	482
O.latipes/T.nigroviridis	1763	305	106	409
G.aculeatus/T.rubripes	2689	2123	652	502
G.aculeatus/T.nigroviridis	3132	1305	262	378
T.rubripes/T.nigroviridis	2536	823	478	564

	N/P + N/N	N/P + P/N	N/P + P/P
D.rerio/O.latipes	2555	2079	2312
D.rerio/G.aculeatus	5231	5077	5481
D.rerio/T.rubripes	5112	4897	5084
D.rerio/T.nigroviridis	4318	4149	4276
O.latipes/G.aculeatus	1873	1910	2545
O.latipes/T.rubripes	2143	1899	2184
O.latipes/T.nigroviridis	2068	1869	2172
G.aculeatus/T.rubripes	4812	3341	3191
G.aculeatus/T.nigroviridis	4437	3394	3510
T.rubripes/T.nigroviridis	3359	3014	3100

	N/P vs N/N	N/P vs P/N	N/P vs P/P	
	p_a-values	p_b-values	p_c-values	p-values *
D.rerio/O.latipes	0.000001	0.000001	0.000001	0.00003
D.rerio/G.aculeatus	0.000001	0.000001	0.000001	0.00003
D.rerio/T.rubripes	0.000001	0.000001	0.000001	0.00003
D.rerio/T.nigroviridis	0.000001	0.000001	0.000001	0.00003
O.latipes/G.aculeatus	0.000001	0.000001	0.000001	0.00003
O.latipes/T.rubripes	0.000001	0.000001	0.000001	0.00003
O.latipes/T.nigroviridis	0.000001	0.000001	0.000001	0.00003
G.aculeatus/T.rubripes	0.000001	0.000001	0.000001	0.00003
G.aculeatus/T.nigroviridis	0.000001	0.000001	0.000001	0.00003
T.rubripes/T.nigroviridis	0.000001	0.000001	0.000001	0.00003

* p-values Bonferroni- corrected

After RepeatMasker

Pairwise species	N/P	N/N	P/N	P/P
D.rerio/O.latipes	1768	764	93	241
D.rerio/G.aculeatus	4692	487	53	453
D.rerio/T.rubripes	4505	579	38	209
D.rerio/T.nigroviridis	3928	371	18	135
O.latipes/G.aculeatus	1559	296	342	996
O.latipes/T.rubripes	1705	451	197	460
O.latipes/T.nigroviridis	1768	304	108	397
G.aculeatus/T.rubripes	2644	2218	625	463
G.aculeatus/T.nigroviridi	3168	1281	267	360
T.rubripes/T.nigroviridis	2569	758	474	538

	**N/P + N/N**	**N/P + P/N**	**N/P + P/P**
D.rerio/O.latipes	2532	1861	2009
D.rerio/G.aculeatus	5179	4745	5145
D.rerio/T.rubripes	5084	4543	4714
D.rerio/T.nigroviridis	4299	3946	4063
O.latipes/G.aculeatus	1855	1901	2555
O.latipes/T.rubripes	2156	1902	2165
O.latipes/T.nigroviridis	2072	1876	2165
G.aculeatus/T.rubripes	4862	3269	3107
G.aculeatus/T.nigroviridi	4449	3435	3528
T.rubripes/T.nigroviridis	3327	3043	3107

	**N/P vs N/N**	**N/P vs P/N**	**N/P vs P/P**	
	p_a-values	p_b-values	p_c-values	p-values *
D.rerio/O.latipes	0.000001	0.000001	0.000001	0.00003
D.rerio/G.aculeatus	0.000001	0.000001	0.000001	0.00003
D.rerio/T.rubripes	0.000001	0.000001	0.000001	0.00003
D.rerio/T.nigroviridis	0.000001	0.000001	0.000001	0.00003
O.latipes/G.aculeatus	0.000001	0.000001	0.000001	0.00003
O.latipes/T.rubripes	0.000001	0.000001	0.000001	0.00003
O.latipes/T.nigroviridis	0.000001	0.000001	0.000001	0.00003
G.aculeatus/T.rubripes	0.000001	0.000001	0.000001	0.00003
G.aculeatus/T.nigroviridi	0.000001	0.000001	0.000001	0.00003
T.rubripes/T.nigroviridis	0.000001	0.000001	0.000001	0.00003

* p-values Bonferroni- corrected
